# Verification of the association of the cycle threshold (Ct) values from HPV testing on Cobas4800 with the histologic grades of cervical lesions using data from two population-based cervical cancer screening trials

**DOI:** 10.1186/s13027-022-00440-4

**Published:** 2022-06-11

**Authors:** Yi Zhang, Hui Du, Aimin Xiao, Wei Zhang, Chun Wang, Xia Huang, Xinfeng Qu, Jianliu Wang, Ruifang Wu

**Affiliations:** 1 Department of Obstetrics and Gynecology, Peking University Shenzhen Hospital, Shenzhen, 518036 People’s Republic of China; 2 Institute of Obstetrics and Gynecology, Shenzhen PKU-HKUST Medical Center, Shenzhen, 518036 People’s Republic of China; 3 Shenzhen Key Laboratory on Technology for Early Diagnosis of Major Gynecologic Diseases, Shenzhen, 518036 People’s Republic of China; 4 Sanming Project of Medicine in Shenzhen Peking University Shenzhen Hospital, Shenzhen, 518036 Guangdong People’s Republic of China; 5 Department of Obstetrics and Gynecology, Peking University People’s Hospital, Beijing, People’s Republic of China

**Keywords:** Cervical cancer, HPV, Screening, Circulating threshold, Viral load

## Abstract

**Objective:**

To verify the association of high-risk human papillomavirus (hrHPV) viral load reflected by cycle threshold (Ct) values from HPV testing on Cobas4800 assay with the histologic grades of cervical lesions via analysis on the combined data from two cervical cancer screening trials and to explore the referability of Ct values in management of the abnormalities from cervical cancer primary screening.

**Methods:**

We analyzed the data from Chinese Multi-Center Screening Trial (CHMUST) and BUJI Cervical Cancer Screening Study Project (BUJI Study). All data to be analyzed in this paper were related to provider-collected samples. One-way ANOVA was performed to compare the Ct values among different histological groups, and Kendall’s tau-b correlation was applied to examine the association between Ct values and cervical lesion grades. The stepwise incidence of CIN2+ and CIN3+ in every 100 HPV positive individuals were calculated according to the descending of the genotype specific Ct values. The highest Ct values related to CIN3+ incidence rate 4% (or 25%) were used as the cutoffs to distinguish low-Ct value cases from the high-Ct value ones.

**Results:**

A total of 1376 women in CHUMUST and BUJI Study who were aged 30–59 and positive of hrHPV for provider-collected samples on Cobas4800 with complete data in terms of the relevant Ct values (CtV) and the histological diagnosis were included for analysis. Our data showed significant difference among different histological grades of cervical lesions in the CtV of hrHPV, HPV16-plus (positive of HPV16 only or HPV16 plus 18 and/or pooled 12-HPV), and pooled 12-HPV (*P* < 0.05). No significant difference was found among different grades of lesions in term of correlated CtV of HPV18-plus (positive of HPV18 only or HPV18 plus pooled 12-HPV) (*P* > 0.05). The CIN2+ or CIN3+ incidence per 100 positives significantly increased corresponding to the descending of the CtV of hrHPV, HPV16-plus and pooled 12-HPV. Compared with high-CtV groups (CtV > 33.2 for hrHPV, CtV > 29.6 for pooled 12-HPV), the relevant risks (RRs) of CIN2+ for hrHPV and pooled 12-HPV positive groups with low-CtV (CtV ≤ 33.2 and ≤ 29.6, respectively) were 3.2 (95%CI 2.18–4.80) and 2.3 (95%CI 1.50–3.45). Similarly, the RRs of CIN3+ for hrHPV and pooled 12-HPV positive groups with low-CtV were 6.5 (95%CI 2.83–14.80) and 2.7 (95%CI 1.15–6.39), respectively. The RRs of CIN2+ for medium- (30.3 < CtV ≤ 37.4) and low- (≤ 30.3) CtV HPV16-plus positives were 5.1 (95%CI 0.68–38.38) and 20.6 (95%CI 2.96–143.92) related to high-CtV (> 37.4) groups, and the CIN3+ incidence in low-CtV value group was nine-fold higher of that in medium-CtV ones [RRs, 9.0 (95%CI 2.89–28.10)]. In comparing with the algorithms of “HPV16-plus/18-plus + cytology ≥ ASCUS for pooled 12-HPV”, triage algorithm “HPV16-plus/18-plus + Ct value ≤ 33.2 for pooled 12-HPV” could achieve a comparable sensitivity of 93.2%.

**Conclusion:**

HPV viral loads reflected by Ct values for hrHPV, HPV16-plus and pooled 12-HPV from Cobas4800 HPV testing were directly associated with the severity of cervical lesions. A lower HPV genotype-specific Ct value prompted a significantly high CIN3+ risk of 4% or higher in women positive of hrHPV, HPV16-plus or pooled 12-HPV, indicating that HPV viral load reflected by Ct values on Cobas4800 may be a promising risk indicator in management of abnormalities from primary cervical cancer screening.

**Supplementary Information:**

The online version contains supplementary material available at 10.1186/s13027-022-00440-4.

## Introduction

Cervical cancer is the fourth leading cause of cancer morbidity and mortality among women worldwide, and 88% of the new cases are from less developed countries [1]. HrHPV is the cause of nearly all cervical cancers. However, only a small portion of women with persistent hrHPV infection could develop into high grade cervical intraepithelial neoplasia (CIN2+) that may potentially progress to cervical cancer if left untreated [2]. More and more studies up to date have suggested that hrHPV viral load play an important role in cervical cancer development [3–5]. The opinion that hrHPV type-specific viral load can be referred as an indicator for triage of hrHPV positives [6, 7] is gradually being accepted.

Cervical cancer is preventable via early diagnose and proper management [8]. HPV testing has been recognized to be an effective primary screening method for population based cervical cancer prevention for its higher sensitivity and negative predictive value comparing with cytological tests in detection of cervical lesions [9]. It is also a cost-effective method when taking the longer screening interval with HPV testing into consideration. However, the prevalence of high-grade lesions is low in HPV-positive women. It is not recommended to refer all the hrHPV-positive women to colposcopy [10]. Therefore, it is anticipated to have effective triage tests to identify women who need immediate colposcopy or treatment from the hrHPV-positives who should just undergo surveillance without requirements for multiple visits. A series of studies have evaluated the effectiveness of viral load in positive triage and indicated the referability in triaging hrHPV-positives via combination of viral load with hrHPV genotypes [11–14].

Several HPV testing assays can report indicators reflecting viral loads in addition to indicating hrHPV infections, such as HC-2 (Qiagen, Gaithersburg, MD, USA) [11, 14], HBRT-H14 (Hybribio, Chaozhou, China) [15], BMRT (BioPerfectus Multiplex Real-Time PCR assay, Taizhou, China) [12], Xpert HPV (Cepheid, Sunnyvale, CA, USA) [16], Cobas4800 (Roche, USA) [13, 17, 18], et al. Among them, Cobas4800 is the only HPV testing assay that has been approved by FDA for primary cervical cancer screening [19]. Cobas4800 detects a total of 14 hrHPV types in three channels: HPV16, HPV18, and pooled 12-HPV (including HPV-31, -33, -35, -39, -45, -51, -52, -56, -58, -59, -66, and -68). Even though the Cobas 4800 is not validated for measuring viral load yet, as a HPV test based on real-time polymerase chain reaction (PCR) technology for amplification and detection, it records the cycle thresholds (Ct values, CtV) of target DNA amplification, which represents the number of amplification cycles needed when the fluorescence signal in each reaction tube reaches the preset threshold in a qPCR assay. CtV is inversely correlated with the log amount of targeted DNA in the specimen [20, 21], meaning that a high CtV indicating a low HPV viral load [16].

Several clinical studies have verified the sensitivity, specificity, and reproducibility of Cobas 4800 for detecting hrHPV genotypes [22–25]. Besides, Cobas 4800 showed good concordance in testing hrHPV with self- and provider-collected samples [26]. Self-sampling for HPV testing may improve the participation in cervical cancer screening programs [27]. If women positive of hrHPV primary testing could be effectively triaged using the HPV genotypes and the Ct values from Cobas 4800 testing, especially for self-collected samples, primary screening and positive triage would be performed with one sampling without needing for multiple visits, which has been demonstrated to be an important adverse element to positive follow-up as well as the inconvenience for participation. However, there has been a limit number of studies on the correlation of CtV in Cobas 4800 with the grades of cervical lesion up to date, with inconsistent conclusions. One study by our team demonstrated a confirmed adverse correlation between CtV of HPV16 and the grades of cervical lesions and reported that the CtV of pooled 12-HPV is significantly lower in women with LSIL or HISL+ than that in women with normal cervix [13, 17]. In another study, virus loads of pooled 12-HPV, but not those of the HPV-16 or -18, were found high in women with cervical lesions [20].

In this study, we reanalyzed the data from two clinical studies completed by our team, Chinese Multi-Center Screening Trial (CHMUST) and BUJI Cervical Cancer Screening Study Project (BUJI Study), to further confirm the potential correlation between hrHPV viral loads and the grades of cervical lesions in women positive of hrHPV via age-grouping, projecting to provide evidences for the referring HPV genotype-specific Ct values from Cobas 4800 to manage the abnormalities from HPV primary screening.

## Materials and methods

### Study design and participants

This study was designed to analyze the data of the hrHPV positives for provider-collected sample on Cobas4800 with recorded Ct values from CHIMUST and BUJI study.

CHIMUST (The Chinese Multi-Center Screening Trial, Registration number: ChiCTR-EOC-16008456) is a multicenter population-based cross-sectional cervical cancer screening study led by our team, in which 10,885 women were screened at 6 sites located in 6 regions nationwide in China from Aug 2016 to Jan 2018. Women enrolled were those who were aged from 30 to 59 years, sexual exposed, and non-pregnant, had not been screened for at least 3 years based on participant report, had no history of hysterectomy and pelvic radiation, and signed the consent form. The trial was approved by the Ethics Committee of the Peking University Shenzhen Hospital (IRB:PUSH2016001) and the Institutional Review Board of Cleveland Clinic (USA) (IRB:15–1549).

Both self-collected vaginal sample and provider-collected endocervical sample were taken from each participant, which were split for HPV testing on Cobas 4800 (Roche, USA) and SeqHPV (BGI, Shenzhen, China). The provider-collected samples were additionally prepared for cytology testing (ThinPrep, Hologic). Cytology slides were analyzed by 2 senior cyto-pathologists from Peking University Shenzhen Hospital (PUSH) and reported according to the Bethesda classification [28], as negative for intraepithelial lesion or malignancy (NILM), atypical squamous cells of undetermined significance (ASC-US), low-grade squamous intraepithelial lesion (LSIL), atypical squamous cells cannot exclude high-grade lesion (ASC-H), atypical glandular cells (AGC), and high-grade squamous intraepithelial lesion or worse (HSIL+).

Participants who were positive of hrHPV from Cobas 4800 assay and/or SeqHPV for both or either self- and/or provider-collected samples were referred to colposcopy. Cytology results were not referred for triage in this study [29]. Colposcopy-directed and random biopsies were taken according to the Preventive Oncology International (POI) protocol [30]. All histology slides were analyzed by a gyn-pathological expert from PUSH, who was blind of HPV and cytology results. Histology results were classified as non-CIN, cervical intraepithelial neoplasia (CIN)1, CIN2, CIN3, adenocarcinoma in situ (AIS), and cancers. Women who were positive of hrHPV for provider-collected sample on Cobas 4800 testing with reported the relevant Ct values (the positive + CtV) were included into the analysis for this study.

BUJI study (BUJI Cervical Cancer Screening Study Project) were conducted in Buji Community, Shenzhen, China, from August 2016 to September 2017. Totally ten thousand (10,000) women who were 19 to 80 years of age, sexually exposed and non-pregnant, had no prior hysterectomy and pelvic radiation, and consented for participation in writing were enrolled for cervical cancer screening with co-test of HPV testing on Cobas4800 and cytology. The study was approved by the Ethics Committee of Peking University Shenzhen Hospital (No. PUSHGYN2015005).

The provider-collected endocervical samples were prepared for hrHPV testing on Cobas 4800 (Roche, USA) and cytology testing using AutoCyte® thin-layer liquid-based technology (TriPath Imaging, Inc). Participants were called back for colposcopy if they were (1) positive of HPV16 and/or HPV18, or (2) positive of pooled 12-HPV plus abnormal of cytology (≥ ASC-US); or (3) negative of hrHPV but Cytology ≥ LSIL. Cytology and histology slides were analyzed by PUSH pathologists, and the interpretations were classified following the same criteria as followed in CHIMUST. Ct values of the 763 women who were positive of HPV from Cobas 4800 testing from the first 7000 women in BUJI study were recorded, of them 705 were at the age matching with CHIMUST (from 30 to 59).

### Cobas 4800 HPV assay

Cobas 4800 HPV assay is a multi-PCR based HPV assay, which simultaneously detects a total of 14 hrHPV types in three channels: HPV16, HPV18, and pooled 12-HPV (including HPV-31, -33, -35, -39, -45, -51, -52, -56, -58, -59, -66, and -68), in addition to a separate channel of β-globin as reference. Cobas 4800 records CtV of each positive HPV channel as well as the reference channel. CtV-cutoff for all the HPV channels were determined by the manufacturers to define as positive (cutoff of 40.5 for HPV16, 40 for HPV 18 and pooled 12-HPV channels). When CtV equals to or lower than the cutoff, we get a positive result and the corresponding CtV is recorded. CtV of negative channels is beyond the default CtV-cutoff and not reported.

In this analysis, hrHPV positive results were categorized according to 3 hierarchies: HPV16-plus, HPV18-plus, and pooled 12-HPV. If not specially indicated, HPV16-plus refers to a result that is positive of HPV16 only or positive of HPV16 plus 18 and/or pooled 12-HPV; HPV18-plus refers to a result that is positive of HPV18 only or positive of HPV18 plus the pooled 12-HPV; while pooled 12-HPV refers to a result that is positive of the pooled 12-HPV only.

### Statistical analysis

Statistical analysis was based on the data of the women who were enrolled in both the two projects, aged of 30–59, positive of hrHPV for provider-collected sample on Cobas 4800 testing with complete data regarding to the relevant Ct values and the histological diagnosis (the analytic group). The main outcome of this analysis was the association of genotype-specific Ct values and the histological grading of the lesions. HPV and histological examination results of the provider-collected samples from CHIMUST and BUJI Study were jointly analyzed. Ct values were described by mean ± standard deviation, and the differences of CtV among histological lesion grades were compared by one-way ANOVA. *Kendall’s tau-b* correlation was used to examine the association between Ct values and cervical lesion grades.

The stepwise incidence of CIN2+ and CIN3+ in every 100 HPV-positives were respectively calculated along with the descending of genotype-specific Ct values. According to the 2019 ASCCP risk-based consensus guidelines for abnormal cervical cancer screening tests and cancer precursors (2019 ASCCP guidelines) [31], the highest CtV related to 4% of the diagnosed CIN3+ cases was referred as the cutoff to differentiate the low-CtV hrHPV and pooled 12-HPV positives from the high-CtV ones. The highest CtV correlated to 4% and 25% of the CIN3+ cases was used as the cutoff to grouping the HPV16 positives into three: low-CtV, medium-CtV, and high-CtV groups. The relative risks (RRs) of CIN2+ or CIN3+ with respective 95% confidence intervals (CI) among low-CtV group were compared with high-CtV ones. *P*-values of diagnostic accuracy between algorithms were obtained by McNemar’s test.

SPSS v.26.0 software (IBM, Armonk, NY, USA) was used for all data analysis in this study with the significance level *P* < 0.05.

## Results

Totally, there were 2051 women in CHUMUST and BUJI Study who were aged 30–59 and positive of hrHPV for provider-collected samples on Cobas4800 with Ct-value reported, including 1346 from CHIMUST and 705 from BUJI Study. After deleting 225 cases from CHIMUST cohort for unreturning for colposcopy and 419 cases from BUJI Study cohort for having no pathology diagnosis because of negative cytology and 31 for unreturning for colposcopy, 1376 women were included in the analytic group, including 1121 from CHIMUST and 255 from BUJI.

Among the positives in the analytic group, the mean age was 44.3 (± 7.64) years. 14.03% (193/1376) were positive of single HPV16, 5.81% (80/1376) of single HPV18, and 71.95% (990/1376) of pooled 12-HPV; 20.49% (282/1376) of them were categorize as HPV16-plus, 7.56% (104/1376) as HPV18-plus (Table 1). 72.60% (999/1376) were pathologically reported as non-CIN, 14.32% (197/1376) as CIN1, 7.70% (106/1376) as CIN2, 5.01% (69/1376) as CIN3, and 0.36% (5/1376) as invasive cervical cancer (Table 1).

**Table 1:** Characteristics of positives included in the analysis

Characteristics	Analytic subjects (n = 1376)
Age (mean ± SD)	44.3 (± 7.64)
HrHPV	1376
HPV16 single infection	193 (14.03%)
HPV18 single infection	80 (5.81%)
Pooled 12-HPV	990 (71.95%)
HPV16-plus	282 (20.49%)
HPV18-plus	104 (7.56%)
Histology	1376
Non-CIN	999 (72.60%)
CIN1	197 (14.32%)
CIN2	106 (7.70%)
CIN3*	69 (5.01%)
Cancer	5 (0.36%)

*Two adenocarcinomas in situ (AIS) cases were included in CIN3 group

HPV16-plus refers to a result that is positive of HPV16 only or positive of HPV16 plus HPV18 and/or pooled 12-HPV; HPV18-plus refers to a result that is positive of HPV18 positive only or positive of HPV18 plus the pooled 12-HPV

*SD* standard deviation, *CIN* cervical intraepithelial neoplasia

The average Ct values of single HPV16, single HPV18, and pooled 12-HPV were 30.5 (± 5.1), 32 (± 5) and 31.1 (± 5.4), respectively. As no significant difference was observed when compared the CtV between any two HPV genotypes and between HPV-16 or -18 single-type infection and multi-type co-infection (*P* > 0.05) (Table 2), analysis on the correlation of Ct values with the histological lesion grades was conducted in groups as HPV16-plus, HPV18-plus, and pooled 12-HPV. No significant difference was either observed in the HPV genotype-specific Ct values among age-group 30–39, 40–49, and 50–59. (Additional file 1: Table S1).

**Table 2:** Distribution of Ct values (CtV) in each HPV genotype

Coinfection genotypes	HPV16		HPV18		Pooled 12-HPV	
	No. of participants	CtV (mean ± SD)	No. of participants	CtV (mean ± SD)	No. of participants	CtV (mean ± SD)
HPV16	193	30.5 (± 5.1)	9	29.9 (± 2.9)	86	31.7 (± 4.7)
HPV18	9	29.8 (± 4.8)	80	32 (± 5.0)	30	31.2 (± 5.3)
Pooled 12-HPV	86	30.9 (± 4.8)	30	32.5 (± 4.3)	990	31.1 (± 5.4)

6 Cases were positive of HPV16 and HPV18 and pooled 12-HPV

Our data showed that Ct values of hrHPV, HPV16-plus and pooled 12-HPV were inversely associated with the severity of cervical lesions (Kendall-Tau-b = -0.205, -0.368 and -0.172, respectively. *P* < 0.001). The CtV of hrHPV and HPV16-plus showed gradually descending along with the upgrading of the histological severities. Detailed grouping analysis showed that (1) Ct values of hrHPV and HPV16-plus correlated to non-CIN and CIN1 were both respectively significant higher than those correlated to CIN2+ and CIN3+ (*P* < 0.05); (2) CtV of pooled 12-HPV correlated to non-CIN was higher than that correlated to CIN1 and CIN2+ (*P* < 0.05); (3) CtV of pooled 12-HPV correlated to CIN1, CIN2+ and CIN3+ showed virtually identical (28.9–29.1); and (4) no significant difference was found among different grades of lesion in terms of correlated CtV of HPV18-plus (*P* > 0.05) (Table 3). When conducting analysis on CtV variability corresponding to the same grade of cervical lesions, we found that (1) there was no significant difference in the CtV of hrHPV or HPV16 between single infection and coinfection with other HPV genotypes respectively in each grade of lesions, and (2) CtV of either HPV single or multiple genotypes infection was significantly lower in higher grade than in lower grade of cervical lesions **(**Table 4). No difference was observed in HPV genotype-specific Ct values corresponding to the same cervical lesion grade among different age groups. The Ct values of hrHPV, HPV16-plus and pooled 12-HPV correlated to CIN2+ were all respectively lower than those correlated to ≤ CIN1 in women at any age group (Table 5).

**Table 3:** Comparison of the Ct values for specific HPV genotype among different grades of cervical lesions

Histology grade	HrHPV		HPV16-plus		HPV18-plus		Pooled 12-HPV	
	No. of participants	CtV (mean ± SD)	No. of participants	CtV (mean ± SD)	No. of participants	CtV (mean ± SD)	No. of participants	CtV (mean ± SD)
Non-CIN	999	31.9 (± 5.1)	156	32.4 (± 4.6)	87	32.1 (± 4.7)	756	31.8 (± 5.2)
CIN1	197	29.6 (± 5.8)	37	31.5 (± 5.4)	10	33.3 (± 5.4)	150	28.9 (± 5.7)^a^
CIN2+	180	28.2 (± 4.8)^a,b^	89	27.0 (± 3.4)^a,b^	7	34.4 (± 5.6)	84	28.9 (± 5.5)^a^
CIN3+	74	27.2 (± 4.2)^a,b^	50	26.3 (± 3.1)^a,b^	2	29.2 (± 3.8)	22	29.1 (± 5.7)

^a^Compared with non-CIN, *P* < 0.05

^b^Compared with CIN1, *P* < 0.05

HPV16-plus refers to a result that is positive of HPV16 only or positive of HPV16 plus 18 and/or pooled 12-HPV; HPV18-plus refers to a result that is positive of HPV18 positive only or positive of HPV18 plus pooled 12-HPV

CIN2+ includes CIN2, CIN3 and cancer. CIN3+ includes CIN3 and cancer

**Table 4:** Comparison of the Ct values of hrHPV/HPV16 between single infection and coinfection

Histology grade	hrHPV single infection		hrHPV coinfection		HPV16 single infection		HPV16 coinfection	
	No. of participants	CtV (mean ± SD)	No. of participants	CtV (mean ± SD)	No. of participants	CtV (mean ± SD)	No. of participants	CtV (mean ± SD)
Non-CIN	936	31.8 (± 5.1)	63	32.8 (± 4.3)	111	32.2 (± 4.7)	45	32.9 (± 4.5)
CIN1	184	29.3 (± 5.8)^a^	13	33.3 (± 4.1)	26	30.8 (± 6)	11	32.9 (± 3.8)
CIN2	84	28.8 (± 5.1)^a^	22	29.1 (± 5.1)	20	28.3 (± 3.9)^a^	19	27.6 (± 3.6)^a,b^
CIN3+	59	27.3 (± 4.5)^a^	15	27.0 (± 2.5)^a,b^	36	26.2 (± 3.4)^a,b^	14	26.6 (± 2.2)^a,b^

^a^Compared with non-CIN, *P* < 0.05

^b^Compared with CIN1, *P* < 0.05

HrHPV single infection refers to a result that is positive of HPV16 only, or HPV18 only, or pooled 12-HPV. HrHPV coinfection refers to a result that is positive of at least two of HPV16, HPV18, and pooled 12-HPV. HPV16 single infection refers to a result that is positive of HPV16 only. HPV16 coinfection refers to a result that is positive of HPV16, plus HPV18 and/or pooled 12-HPV

**Table 5:** Comparison of the genotype specific Ct values in each histology grades among different age group

Histology grade	HrHPV		HPV16		Pooled 12-HPV	
	No. of participants	CtV (mean ± SD)	No. of participants	CtV (mean ± SD)	No. of participants	CtV (mean ± SD)
≤ *CIN1*						
30–39	344	31.6 (± 5.5)	76	33.3 (± 4.7)	237	31.1 (± 5.7)
40–49	513	31.3 (± 5.3)	74	31.4 (± 4.8)	409	31.2 (± 5.4)
50–59	339	31.8 (± 4.9)	43	31.7 (± 4.7)	260	31.7 (± 5.1)
*CIN2*+						
30–39	60	28.4 (± 5.4)^a^	38	27.4 (± 4.3)^a^	21	29.8 (± 6.7)
40–49	82	28 (± 4.3)^a^	36	26.7 (± 2.8)^a^	42	28.6 (± 4.8)^a^
50–59	38	28.4 (± 4.8)^a^	15	27 (± 2.4)^a^	21	28.7 (± 5.5)

^a^Compared with ≤ CIN1, *P* < 0.05

≤ CIN1 includes Non-CIN and CIN1; CIN2+ includes CIN2, CIN3 and cancer

**Fig. 1:**
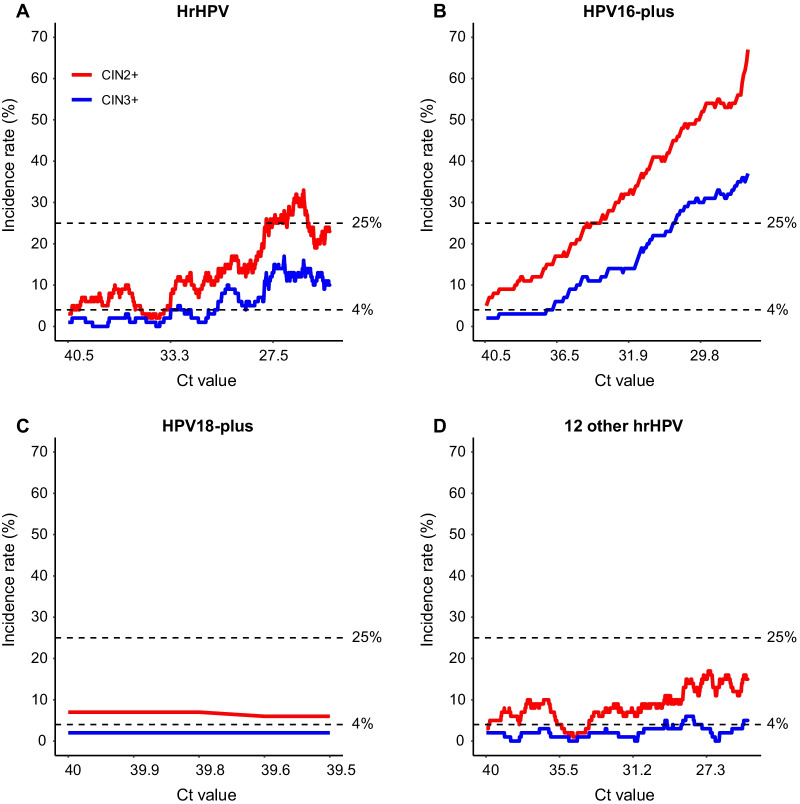
The tendency of incidence rate of CIN2+ or CIN3+ in HPV positives. The curves represent the stepwise incidence rates of CIN2+ (red) or CIN3+ (blue) in every 100 hrHPV positive cases (**A**), HPV16-plus positives (**B**), HPV18-plus positives (**C**) and pooled 12-HPV positives (**D**). The x-axis shows the highest Ct value of each 100 individuals in decreasing order, and the y-axis shows the incidence rate (%)

Analysis also showed that the CIN2+ or CIN3+ incidence per 100 positives significantly increased corresponding to the descending of the Ct values of hrHPV, HPV16-plus and pooled 12-HPV (Fig. 1). The highest Ct values of hrHPV, HPV16-plus and pooled 12-HPV correlated to 4% of CIN3+ incidence in the positives were 33.2, 37.4, and 29.6, respectively. When choosing CtV of HPV16-plus to be 30.3 or lower, the correlated CIN3+ incidence in HPV16-plus positives was 25% or higher. According to the 2019 ASCCP risk-based consensus guidelines for abnormal cervical cancer screening tests and cancer precursors (2019 ASCCP guidelines) [31], Ct values of 33.2 and 29.6 were used as the cutoffs to distinguish high-CtV (CIN3 + risk < 4%) cases from low-CtV (CIN3 + risk ≥ 4%) ones among women positive of hrHPV and pooled 12-HPV. Compared with high-CtV groups, the relevant risks (RRs) of CIN2+ for hrHPV and pooled 12-HPV positive groups with low-CtV were 3.2 (95%CI 2.18–4.80) and 2.3 (95%CI 1.50–3.45). Similarly, the RRs of CIN3+ for hrHPV and pooled 12-HPV positive groups with low-CtV were 6.5 (95%CI 2.83–14.80) and 2.7 (95%CI 1.15–6.39). When using CtV 37.4 and 30.3 as the cutoffs to group HPV16-plus positives into high-CtV, medium-CtV, and low-CtV groups, only one CIN2+ and no CIN3+ was included in high-CtV group. The RRs of CIN2+ for medium- and low-CtV HPV16-plus positives were 5.1 (95%CI 0.68–38.38) and 20.6 (95%CI 2.96–143.92) related to high-CtV groups, and the CIN3+ incidence in low-CtV groups was nine-fold higher of that in medium-CtV ones [RRs, 9.0 (95%CI 2.89–28.10)] (Table 6).

**Table 6:** Relative risks (RRs) of CIN2+ and CIN3+ for low-Ct HPV positive cases relative to high-Ct HPV positive cases

**Factors**	Absolute risks for CIN2+ (%)	Relative risks for CIN2+ (95% CI)	Absolute risks for CIN3+ (%)	Relative risk for CIN3+ (95% CI)
hrHPV	13.1 (180/1376)		5.4 (74/1376)	
CtV > 33.2	5.4 (27/500)	1	1.2 (6/500)	1
CtV ≤ 33.2	17.5 (153/876)	3.2 (2.18–4.80)	7.8 (68/876)	6.5 (2.83–14.80)
HPV16-plus	31.6 (89/282)		17.7 (50/282)	
Ct > 37.4	2.4 (1/41)	1	0 (0/41)	NA
30.3 < CtV ≤ 37.4	12.5 (11/88)	5.1 (0.68–38.38)	3.4 (3/88)	1
CtV ≤ 30.3	50.3 (77/153)	20.6 (2.96–143.92)	30.7 (47/153)	9.0 (2.89–28.10)
Pooled 12-HPV	8.5 (84/990)		2.2 (22/990)	
CtV > 29.6	5.7 (34/601)	1	1.3 (8/601)	1
CtV ≤ 29.6	12.9 (50/389)	2.3 (1.50–3.45)	3.6 (14/389)	2.7 (1.15–6.39)

Absolute risks for CIN2+ or CIN3+ is the number of subjects with CIN2+ or CIN3+ /number of subjects with positive test results

*CI* confidence interval, *NA* not applicable

The consistency between HPV positive results with low-CtV (CtV ≤ 33.2 for HrHPV, HPV16-plus/18-plus+ CtV ≤ 33.2 for pooled 12-HPV) and specific histology grades were showed in Table 7. Interestingly, the consistent rate significantly increased along with the historical cervical lesion grading up (*P* < 0.001), indicating that the higher the lesion is the more consistent the low-CtV is with the lesion. We then tried to construct algorithms for positive triage base on CtV and compared them with multiple recommended algorithms, and demonstrated that, in comparing with the algorithms of “HPV16-plus/18-plus+ cytology ≥ ASCUS for pooled 12-HPV”, making “HrHPV CtV ≤ 33.2” as the triage algorithm (algorithm E) could achieve a lower sensitivity of 91.9% and lower specificity of 37.9% for CIN3+ , while making “HPV16-plus/18-plus+ CtV ≤ 33.2 for pooled 12-HPV” as triage algorithm (algorithm F) could achieve a comparable sensitivity of 93.2% and lower specificity of 28.4% for CIN3+ , with 72.8% of colpo referral rate (Table 8).

**Table 7:** The consistency between low-CtV hrHPV positives and histology grades

	Total(%)	Non-CIN(%)	CIN1 (%)	CIN2+ (%)	CIN3+ (%)
HrHPV	1376	999	197	180	74
HPV16-plus/18-plus	386 (28.1%)	243 (24.3%)	47 (23.9%)	96 (53.3%)	52 (70.3%)
HrHPV CtV ≤ 33.2	876 (63.7%)	584 (58.5%)	139 (70.6%)	153 (85.0%)	68 (91.9%)
HPV16-plus/18-plus+ CtV ≤ 33.2 for pooled 12-HPV	1001 (72.7%)	681 (68.2%)	159 (80.7%)	161 (89.4%)	69 (93.2%)

**Table 8:** Comparison of different screening strategies

Algorithms	CIN2+ (n = 180)		CIN3+ (n = 74)		Colposcopy referral rate%	Cytology testing rate%	Colposcopies to detect 1 CIN2+ /CIN3+
	Sen%	Spe%	Sen%	Spe%			
A. HPV16-plus/18-plus	53.3*	75.7*	70.3*	74.3*	28.1	NA	4.0/7.4
B. Cytology ≥ ASCUS	80.0*	66.7*	97.3	63.9*	39.4	100	3.8/7.5
C. Cytology ≥ LSIL	66.1*	84.7*	89.2*	81.9*	22.0	100	2.6/4.6
D. HPV16-plus/18-plus+ cytology ≥ ASCUS for pooled 12-HPV	90.6	49.6	100	46.9	55.6	72.0	4.7/10.3
E. HrHPV CtV ≤ 33.2	85.0	39.5*	91.9*	37.9*	63.7	NA	5.7/12.9
F. HPV16-plus/18-plus+ CtV ≤ 33.2 for pooled 12-HPV	89.4	29.7*	93.2	28.4*	72.8	NA	6.2/14.5

hrHPV positive, n = 1375

*Compared with algorithm D, *P* < 0.05

One woman with invalid cytology result was excluded in the analysis

## Discussion

Our results showed that the Ct values of hrHPV, HPV16-plus and pooled 12-HPV genotypes were inversely associated with the severity of cervical intraepithelial neoplasia and the risk of CIN2+ or CIN3+. The viral load of HPV18-plus reflected by CtV had no such correlation with the degree of cervical lesions or the risk of CIN2+/CIN3+. This result is consistent with the prior analysis based on BUJI study [17], showed a stable relationship between HPV Ct values from Cobas4800 testing and the grades of cervical lesions in different populations.

Linear descending with grading-up of the histological cervical lesions were shown in the CtV for HPV16-plus but not in that for pooled 12-HPV. The explanation for the nonlinear correlation between CtV for pooled 12-HPV and cervical lesion grades may possibly lay on the fact that Cobas4800 assay makes the 12 hrHPV genotypes (31, 33, 35, 39, 45, 51, 52, 56, 58, 59, 66, and 68) in pool which may result in different pathogenic activity to a specific precancer lesion and therefore impacts the correlation of the CtV to the cervical lesions, and it is unable to distinguish the Ct-value for each single-genotype infection from that for multi-genotype infection. Early studies had confirmed the differences in the pathogenicity, either viral load related or not, of different hrHPV genotypes. One study compared the positive predictive value (PPV) of 13 hrHPV genotypes for CIN2+ or CIN3+ and found that the cervical precancerous lesion risks were significantly different among these hrHPV genotypes; the alpha-9 species (including HPV16, 31, 33, 35, 52, 58) are phylogenetically similar to HPV16 and closely associated with precancerous lesions; and the top three genotypes (HPV16/33/31) with the highest CIN2+ /CIN3+ risk were all within the alpha-9 species [32]. Another study used qPCR to test the relationship between pathological gradings of cervical lesions and the viral load of 14 hrHPV genotypes and reported a positive correlation in some hrHPV genotypes including HPV 16, 18, 31, 33, 51, 52, 53, and 58, but no correlation in others [33]. An early study from our team also reported inconsistent relationship between viral load and cervical lesion grades in different hrHPV genotypes, for example, the CtV reflected viral loads for HPV16, 33 and 58 were significantly different among all grades of cervical lesions, whereas the viral load of HPV18, 31, 35, 39, 45, 51, 52, 56, 59, 66 and 68 were not predictive for cervical lesion severity [12]. On the other hand, multi-genotype infection may interfere the correlation between cervical lesion grades and Ct values for pooled 12-HPV. Adcock et al*.* reported that coinfection with lower hierarchy HPV genotypes added little risk for CIN2+ or CIN3+, [32] indicating that the cervical lesions related to multi-HPV-genotypes coinfection could be mainly driven by the higher risk subtypes, and the contribution of lower risk HPV types to the cervical lesion is negligible. Our study verified this view by showing no significant difference in HPV16 CtV of the same lesion grade between HPV16 single infection and HPV16 coinfection with other genotypes. This result suggests that, when pooling the 12 HPV genotypes to analyze the relationship between viral load and disease, the significant difference in Ct value among different grades of lesions could be mainly driven by the higher risk subtypes. The coinfection of lower risk subtypes could lead to a high overall viral load (showed a lower CtV) but did not upgrade the pathogenicity. Therefore, testing each hrHPV genotype and reporting the relevant viral load separately is needed to verify the relationship between each of the 12 hrHPV and the grade of cervical lesions.

Our work showed no correlative descending of HPV18-plus Ct values corresponding to the cervical lesions upgrading. The CtV for CIN3+ related HPV18-plus was not significantly different than that for CIN2+ related HPV18-plus, although little lower was observed. The small number of cases with HPV18-plus related CIN2+ (7 patients) may bias the finding in this study. In addition, several previous studies demonstrated the controversial correlation of the HPV18 viral loads and the cervical lesion grading. Some authors reported viral load increasing along with upgrading of the HPV18-postivie cervical lesions [34] or comparatively higher PPV for CIN2+ or CIN3+ in higher viral load HPV18 positive women [32]. Other reported no correlation between HPV18 viral load and the lesion severity [12, 13, 18, 35, 36]. Further studies involving more HPV18 positive women may be needed to demonstrate the correlation between viral load for HPV18 and the grade of cervical lesions.

In view of the correlation of HPV Ct values to the cervical lesion grades and its predictive value on CIN2+ or CIN3+ lesions, the viral loads reflected by Cobas4800 CtV may potentially be a referable indicator for CIN risk in management of abnormalities from primary cervical cancer screening. However, how to refer HPV Ct values to achieve the favorable management is still a great challenge. Previous studies mainly focused on the triage value of non-16/18 hrHPV viral load, and mostly based on the quartile CtV of the tested population [13, 18] or the reported range of HPV test [3, 11, 14] to determine the viral load cutoffs for triage. Earlier studies from our team demonstrated that HPV16/18 genotypes combined with an appropriate cutoff of CtV for the pooled 12-HPV could be a promising triage for HPV-positive women, which had been evidenced by the similar sensitivity and slightly lower specificity when compared with the widely used triage algorithm of “HPV16/18 and cytology ≥ ASC-US of pooled 12-HPV” [13, 18].

Our work showed a strong correlation between CtV of HPV16 and the risk of cervical lesions. Since 2019 ASCCP guidelines recommends management of cervical cancer screening abnormalities based on the CIN3+ risk yielding from any combination of history and current test results [31], and our results showed a gradually rise of CIN3+ risks according to the descending of HPV genotype-specific Ct values, it is feasible to refer the viral load of HPV16-plus to achieve an effective HPV-positive management. For every 100 hrHPV, HPV16-plus or pooled 12-HPV positive women, the risk of CIN3+ can reach to 4% or higher (even 25% or higher in HPV16-plus positive women) when the CtV descends to a certain value, which demonstrates the value of genotype-specific (especially HPV16) Ct values in the management of abnormalities from cervical cancer primary screening.

Our study also demonstrated that triage based on HPV genotyping together with HPV genotype-specific CtV was more effective in triage than using CtV as the only indicator for triage, although there was no significant difference among the average CtV of each genotype of HPV (Table 2). When making the overall HPV genotypes as a whole (hrHPV), women with CtV ≤ 33.2 shows high risk for CIN3+ (≥ 4%) and need immediate management according to the 2019 ASCCP guidelines [31]. When making HPV genotyping and HPV genotype-specific CtV as combined risk factors, the CtV-cutoff for HPV16-plus to differentiate CIN3+ high risk patients from the low risk ones was 37.4, while the cutoff for pooled 12-HPV was 29.6 (Fig. 1). It is obvious that the CIN3+ risk of HPV16-plus positives is much higher than that of women positive of pooled 12-HPV under the same CtV or viral load. Using the same CtV cutoff (33.2) to triage patients positive for HPV16-plus and pooled 12-HPV may lead to 17.0% (41/241) of patients who are positive of HPV16-plus with high risk for CIN3+ not to be diagnosed and treated in time, and 37.6% (226/601) of those who positive of pooled 12-HPV with low risk for CIN3+ to be overtreated.

This study showed the predictive effect of HPV16-plus Ct values for CIN3+ risk, which may be socioeconomically high valued in hierarchical management of HPV16-plus positives. When patients with 4% or higher CIN3+ risk are referred for colposcopy, 14.5% (41/282) of HPV16 positives wouldn’t need immediate colposcopy and no CIN3+ lesion would be missed. More importantly, the low-CtV (≤ 30.3) of HPV16-plus indicates a very high CIN3+ risk (≥ 25%) where expedited treatment or colposcopy is acceptable according to 2019 ASCCP guidelines. No correlation was observed between CtV for HPV18-plus and cervical lesions in this study. Considering the relatively low infection rate of HPV18, its high correlation with adenocarcinomas [37] and inferior survival [38–40], and its ability to induce malignant tumors at low level of infections [41, 42], it might be not recommendable to triage HPV18 positives in referring of its CtV. Since 71% of invasive cervical cancers are caused by HPV16/18 [37], women positive of pooled 12-HPV have a much lower risk for high grade cervical lesions or cancer and effective triage is necessary for these women. When CIN3+ risk ≥ 4% is used as a triage indicator, 60.7% (601/990) of pooled 12-HPV positives wouldn’t need referral for colposcopy, which could effectively reduce the clinical pressure for positive management and the psychological burden of patients. However, this triage algorithm may lead to 36.4% (8/22) of the pooled 12-HPV related CIN3+ patients be non-diagnosed. Because of this, triage algorithm with “HPV16-plus/18-plus+ CtV ≤ 29.6 for pooled 12-HPV” showed a lower sensitivity for CIN2+/CIN3+ (data not shown) compared to “HPV16-plus/18-plus+ CtV ≤ 33.2 for pooled 12-HPV” and “HPV16-plus/18-plus+ cytology ≥ ASCUS for pooled 12-HPV” in this study. It is recommended to determine the CtV-cutoff for each HPV genotype separately or combine other risk factors to optimize the management of pooled 12-HPV positive women.

Our study reviewed the data from two large clinical studies involving 1376 HPV positives from six diverse provinces of China, which rendered the results applicable to the general population in various settings. However, there are several limits in our study. Firstly, the viral load in the sample may be influenced by the quality of the sample. In order to reduce the bias caused by the sample cellularity difference in each sample, we obtained cervical exfoliated cell samples by a standardized sampling procedure of removing cervical secretions and rotating brush 3 times. By using this method, several studies published by different teams have all confirmed the clinical value of HPV viral load in triaging of HPV primary screening positives [3, 11, 13, 14, 43, 44], assessing the lesion risk [43, 45], and predicting the prognosis of CIN [44–46], and none of them indicated the need for detecting of the number of cells in the sample. Besides, as a cross-sectional study, we cannot predict the long-term cervical lesion risk of women with different HPV genotype-specific Ct values. More large-scale long-term prospective cohort studies are required to validate the long-term risk of CIN2+/CIN3+ corresponding to different genotype-specific Ct values. Finally, Cobas4800 reports the result of 12 non-16/18 hrHPV genotypes in pool. Their pathogenicity differences and multi-genotype coinfection may interfere with our results. We need to introduce other HPV assays (such as SeqHPV or BMRT) which provide results of each subtype to verify the relationship between cervical lesion grades and Ct value of these 12 hrHPV genotypes (especially alpha-9 species).

## Conclusion

In conclusion, HPV viral loads reflected by Ct values of hrHPV, HPV16-plus and pooled 12-HPV from Cobas4800 HPV testing were associated with the severity of cervical lesions. The Ct values of hrHPV and HPV16-plus, but not pooled 12-HPV, showed linear descendance with the histological upgrading of the cervical lesions. Ct values of HPV18-plus showed no correlation with the grades of cervical lesions. A lower HPV genotype-specific Ct value prompted a significantly high CIN3+ risk of 4% or higher in women positive of hrHPV, HPV16-plus or pooled 12-HPV, this CIN3+ risk can even get up to 25% or higher in HPV16-plus positives with low-CtV, indicating that HPV viral load reflected by Ct values on Cobas4800 may be a promising risk indicator in management of abnormalities from primary cervical cancer screening.

## Supplementary Information

**Additional file 1: Table S1.** Comparison of the Ct values of specific HPV genotype among different age groups.

## Data Availability

All data generated or analyzed during this study are included in this article. The datasets used and/or analyzed during the current study are available from the corresponding author on reasonable request.
