# Supplementary material for: Verification of the association of the cycle threshold (Ct) values from HPV testing on Cobas4800 with the histologic grades of cervical lesions using data from two population-based cervical cancer screening trials
Source: Infect Agent Cancer. 2022 Jun 11;17:27. doi: 10.1186/s13027-022-00440-4 (PMC9188717; doi:10.1186/s13027-022-00440-4)
Supplement: Supplementary file 1 — Additional file 1: Table S1. Comparison of the Ct values of specific HPV genotype among different age groups. [file 13027_2022_440_MOESM1_ESM.docx]

**Supplementary table1.** Comparison of the Ct values of specific HPV genotype among different age groups

| Age | HrHPV | |  | HPV16-plus | |  | HPV18-plus | |  | 12 other hrHPV | | |
| --- | --- | --- | --- | --- | --- | --- | --- | --- | --- | --- | --- | --- |
|  | No. of participants | CtV  (mean±SD) |  | No. of participants | CtV  (mean±SD) |  | No. of participants | CtV  (mean±SD) |  | No. of participants | | CtV  (mean±SD) |
| Total | 1376 | 31.1(±5.3) |  | 282 | 30.6(±5) |  | 104 | 32.3(±4.9) |  | 990 | 31.1(±5.4) | |
| 30-39 | 404 | 31.1(±5.6) |  | 114 | 31.3(±5.3) |  | 32 | 31.6(±5.4) |  | 258 | 30.9(±5.8) | |
| 40-49 | 595 | 30.9(±5.3) |  | 110 | 29.8(±4.8) |  | 34 | 32.8(±5.1) |  | 451 | 31(±5.4) | |
| 50-59 | 377 | 31.4(±5) |  | 58 | 30.5(±4.7) |  | 38 | 32.5(±4.1) |  | 281 | 31.5(±5.2) | |
